# Reinforcement of Tobacco Control and Reduction in Medical Utilization for Asthma in Taiwan: A Population-Based Study

**DOI:** 10.3390/ijerph16203950

**Published:** 2019-10-17

**Authors:** Huang-Ju Liang, Ming-Jiuan Wu, Jih-Shuin Jerng, Chiang-Hsing Yang

**Affiliations:** 1 Department of Health Care Management, National Taipei University of Nursing and Health Sciences, Taipei 108, Taiwan; 020096@ntuh.gov.tw (H.-J.L.); yangch@ntunhs.edu.tw (C.-H.Y.); 2 Center for Quality Management, National Taiwan University Hospital, Taipei 100, Taiwan; 3 Department of Business and Management, Ming Chi University of Technology, New Taipei City 243, Taiwan; mjgrace@mail.mcut.edu.tw; 4 Department of Internal Medicine, National Taiwan University Hospital, Taipei 100, Taiwan

**Keywords:** tobacco ban, smoking, asthma, healthcare utilization

## Abstract

Environmental air quality can affect asthma control and the development of overt asthmatic manifestations. In this population-based study, we investigated the effect of reinforcing a smoking ban in Taiwan through the enactment of the Tobacco Hazards and Prevention Act (THPA) on healthcare utilization rate by asthmatics. Analysis was performed based on data relevant to non-hospitalized asthmatic patients with insurance claims between 2005 and 2013 from the National Health Insurance Research Database of Taiwan, reported data on Asian dust storms, and penalty rates for violations of the tobacco ban. Poisson regression showed that the risk for outpatient visits for asthma was lower after enactment of the THPA (RR = 0.98, 95% CI = 0.98–0.99), with a yearly trend of a reduced risk (RR = 0.99, 95% CI = 0.99–1.00), also lower in geographic regions with medium (RR = 0.79, 95% CI = 0.79–0.80) and high (RR = 0.91, 95% CI = 0.91–0.92) penalty rates. Subgroup analysis showed that asthma visit rates were reduced in both male and female groups after the enactment of the THPA. The risk of an asthma ER visit was increased after the enactment of the amended THPA (RR = 1.07, 95% CI = 1.05–1.09), although the yearly trend was not significant (RR = 1.00, 95% CI = 1.00–1.00). The risk of emergency room visits for asthma was significantly reduced in regions with medium (RR = 0.68, 95% CI = 0.68–0.69) and high (RR = 0.75, 95% CI = 0.74–0.76) penalty rates. Subgroup analysis showed that the visit rates were similar in both male and female groups. The effectiveness of reinforcing the smoking ban warrants further policies aimed at further reducing passive smoking.

## 1. Introduction

The interplay between asthma burden and smoking control is an important issue. Asthma and smoking interact to cause more severe symptoms and difficulty in treatment [1], and smokers with asthma may develop worse asthma control than nonsmokers with asthma [2]. Smoking cessation may improve symptoms and lung function of asthmatic patients; however, the low rates of smoking cessation highlight the need for better strategies to manage this issue [3]. Tobacco smoke is a well-known risk factor for the development of fixed airflow limitation that cannot be controlled with standard asthma medications. In addition, people with asthma may have an accelerated decline in lung function and develop airflow limitation that is not fully reversible, which is often associated with more persistent dyspnea. Moreover, exposure to cigarette smoke has been reported to be an independent risk factor for fixed airflow limitation [4], and tobacco smoke has also been reported to be a significant risk factor for asthma exacerbation in adults and children aged 5 years and younger within months after exposure [5]. Strongly encouraging people with asthma to avoid environmental smoke exposure is an important non-pharmacologic intervention [5].

One strategy to reduce the effect of smoking on asthma is the implementation of smoking restriction legislation such as smoking bans. In a meta-analysis study, Tan and Glantz reported that hospitalization and mortality rates due to respiratory diseases (wheezing) were significantly reduced after smoking bans had been implemented in workplaces, restaurants, and bars [6]. In Scotland, the passage of smoke-free legislation in 2006 was associated with a subsequent reduction in the rates of respiratory diseases in populations with non-occupational exposure to environmental tobacco smoke [7]. In addition, a comprehensive state-wide smoking ban in Arizona resulted in reduced hospitalizations for major diseases, including asthma [8]. A study from Hong Kong evaluated 75,870 hospitalized children (<18 years) with lower respiratory tract infections over a 9-year period, and found that the average hospitalization rate fell by 13.9% per year after smoke-free legislation had been implemented in public places [9]. However, there are thought to be more non-hospitalized cases than hospitalized cases [10], and few studies have investigated the use of healthcare resources in emergency room and outpatient settings. 

In January 2009, the government of Taiwan enacted the amended 1997 Tobacco Hazards and Prevention Act (THPA) that extended smoke-free areas to include almost all enclosed workplaces and public places, adding graphic health warnings to cigarette packages, entirely banning tobacco advertisements, promotion, and sponsorship, and increasing tobacco taxes [11]. This revision of the amended act was based on the recommendations of the World Health Organization (WHO) Framework Convention on Tobacco Control (FCTC) [12], and evidence of its impact on smoking cessation has been reported [13]. In this study, we aimed to investigate the impact of the implementation of the amended THPA on the healthcare utilization associated with asthma in Taiwan.

## 2. Materials and Methods

### 2.1. Design, Data Source and Study Population

This was a population-based, retrospective analysis conducted to assess changes in healthcare utilization by asthmatic patients in Taiwan after the enactment of the amended THPA. This observational intervention study used secondary data from the National Health Insurance Research Database (NHIRD) of Taiwan from 1 January 2005 to 31 December 2013 to explore and analyze changes in the utilization of emergency medical resources for asthma and respiratory diseases (International Classification of Diseases, Ninth Revision, Clinical Modification, ICD-9-CM 415, 428, 490–494, 496). The NHIRD contained outpatient visits, hospital stays, prescription, and disease and vital status for 99% of the country’s population. The datasets this study accessed after approval were protected under the government regulations; no public access was allowed. This study was approved by the National Taipei University of Nursing and Health Sciences (201510EM006), and the need for informed consent from the patients was waived.

### 2.2. Variables and Outcome Measures

#### 2.2.1. Primary Outcome

We used asthma visit rates, including outpatient and emergency room (ER) visits, as the primary outcome measure. Population-based visit rates were defined as the ratio of the number of outpatient visits and ER for asthma visits per month to the total population in Taiwan per month. Numerators were obtained according to the number of monthly outpatient and ER claims, with documented asthma (ICD-9-CM 493) included in the first three principle diagnoses. Denominators were obtained from the government census data. For outpatient claims, we excluded those with requests for refill prescriptions without physician visits. In Taiwan, patients can refill their prescriptions twice without visiting a physician after the first visit. This was identified by the same serial number of outpatient visits.

#### 2.2.2. Independent Variables

We chose two variables as the main independent variables: (1) the period before or after the enactment of the amended THPA in January 2009; and (2) the penalty rates of the county where the healthcare insurance claims were issued after January 2009, defined as the number of penalties divided by the number of audits, and categorized into three groups as high (11.1‰–30.0‰), medium (6.1‰–11.0‰), and low (0.0‰–6.0‰), based on the penalty rate in each county. We compared time periods before the THPA was enacted (2005–2008) and after the THPA was enacted (2009–2013), a total of 9 years. After expanding the scope of smoke-free areas, the health authorities of all counties and cities strengthened the inspection and punishment of illegal smokers and reported the numbers of inspection and punishment to the Ministry of Health and Welfare. According to the act, fines would be issued to both individuals and businesses for violations.

#### 2.2.3. Control Variables

The following control variables were obtained from the NHIRD: (1) gender of the patients with valid claims for health insurance, (2) age of the patients with valid claims for health insurance, and (3) months with documented Asian dust storms to correlate with the months of claims. Asian dust storms have been shown to be detrimental to airway diseases such as childhood asthma in Asian countries [14,15]. We retrieved data regarding the periods of Asian dust storms from 2005 to 2013 from the webpage of the Environmental Protection Administration in Taiwan [16].

#### 2.2.4. Missing Data

We excluded claims with any missing data on identification number, birth date or gender.

### 2.3. Statistical Analysis

We performed descriptive and inferential analyses. For the descriptive statistics, we summarized the demographic features before and after the implementation of the amended THPA, and the severity of the penalty after the implementation of the amended THPA. Continuous variables were expressed as mean ± SD, while categorical variables were expressed as a number (%). For inferential statistics, we analyzed differences and contributing factors related to outpatient and ER visits before and after the implementation of the amended THPA. All analyses were performed using SAS 9.4 statistical software (SAS Institute Inc., Cary, NC, USA). Dependent variables, control variables, and interference factors were included as dummy variables. A Poisson regression model was used to analyze the number of occurrences of monthly asthmatic emergency visits in the first 4 years and 5 years after the implementation of the amended THPA. Months with Asian dust storm were also treated with dummy variables. Subgroup analysis for different genders, and analyses based on the period with or without Asian dust storms were performed with stratified analysis.

## 3. Results

### 3.1. Demographic Data

**Figure 1:**
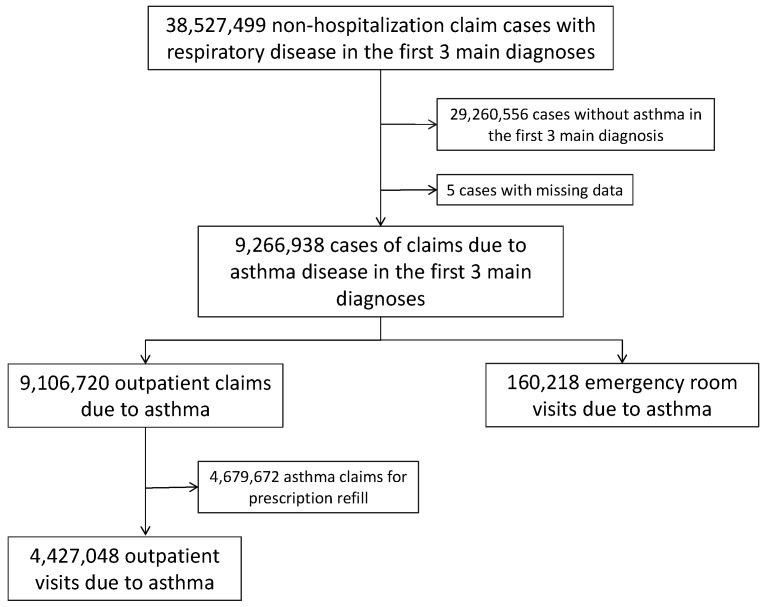
Flow diagram of patient inclusion and exclusion in this study.

From 2005 to 2013, there were 38,527,499 non-hospitalization claims for respiratory diseases. Figure 1 shows the flow diagram of the patient inclusion and exclusion process.

Table 1 shows the number of outpatient and emergency department visits according to classifications. There were 4,427,048 outpatient visits for asthma (ICD-9-CM 493), including 1,919,687 visits in the pre-implementation period and 2,507,361 in the postimplementation period. Male patients (2,543,457, 57.45%) made more visits than female patients. The average age of the visiting patients was 34.11 ± 33.56 years. There were higher percentages of visits toward the extremes of age: 47.07% of outpatient visits were in those aged 0–9 years, and 27.92% were in those aged ≥70 years. During the study period, there were 160,218 ER visits for asthma, including 71,211 visits in the pre-implementation period and 89,007 in the postimplementation period. Male patients (99,501, 62.10%) made more visits than female patients. The average age of the visiting patients was 32.17 ± 33.35 years. There were higher percentages of visits toward the extremes of age: 46.84% of ER visits were in those aged 0–9 years and 27.02% were in those aged ≥70 years (Table 1).

According to the webpage of the Environmental Protection Administration, the air quality was affected by Asian dust storms in the following months: March, November, and December of 2005; March and April of 2006; January, April, and December of 2007; March of 2008; April and December of 2009; March, April, and December of 2010; April and May of 2011; and March of 2012. The air quality was not affected by Asian dust storms at any time in 2013 [16].

Analysis of the penalty rates for violating the smoking ban showed that the annual average penalty rate was low (0.0‰–6.0‰) in seven cities and counties, medium (6.1‰–11.0‰) in eleven cities and counties, and high (11.1‰–30.0‰) in four cities and counties (Table 2). Of the outpatient claims in 2009–2013, 40.35% were issued in the cities and counties with a high average annual penalty rate, whereas 31.80% were in those with a medium penalty rate, and 27.85% were in those with a low penalty rate. Of the claims for an ER visit in 2009–2013, 35.36% were issued in the cities and counties with a high average annual penalty rate, whereas 33.17% were in those with a medium penalty rate, and 31.46% were in those with a low penalty rate (Table 2).

### 3.2. Effect of Reinforcing the Smoking Ban on Asthma Visit Rates

Crude estimates of outpatient asthma visit and emergency department rates and further categorization by control variables are shown in Table 3. The results showed that the overall crude visit rates were similar (1.75‰ vs. 1.80‰) during the two periods before and after the enactment of the amended THPA. Subgroup analysis showed that visit rates were increased in both male and female groups after the enactment of the THPA, and in younger patients (both 0–9 year and 10–19 year groups), but were decreased in adult and older groups (20–59 year, 60–69 year, and ≥70 year groups). The crude visit rates were also the highest in counties and cities with a low penalty rate for smoking. The crude visit rates were similar (0.06‰ vs. 0.06‰) during the periods before and after the enactment of the amended THPA. Subgroup analysis showed that the visit rates were similar in both male and female groups, and were increased in younger patients (both 0–9 year and 10–19 year groups), after the enactment of the THPA, but were decreased in older groups (60–69 years and ≥ 70 year groups). The crude visit rates were also the highest in counties and cities with a low penalty rate for smoking.

The results of multivariate Poisson regression analysis for changes in outpatient and ER asthma visit rates are summarized in Table 4, respectively. After controlling for Asian dust storms and demographic characteristics, we found that the postimplementation period had a reduced risk of outpatient asthmatic visits (RR = 0.98, 95% CI = 0.98–0.99), with a yearly trend of a reduced risk (RR = 0.99, 95% CI = 0.99–0.99). Geographic regions with medium and high penalty rates for smoking also had a lower risk of an outpatient visit. However, Poisson analysis showed that the risk of an asthma ER visit was increased after the enactment of the amended THPA (RR = 1.07, 95% CI = 1.05–1.09), although the yearly trend was not significant (RR = 1.00, 95% CI = 1.00–1.00). Geographic regions with medium and high penalty rates for smoking also had a lower risk of an ER visit. Asian dust storms were significantly associated with outpatient visits of the same months (RR = 1.03, 95% CI = 1.02–1.03). However, ER visits were not significant affected by dust storms (RR = 1.02, 95% CI = 1.00–1.03).

We also performed stratified regressions with subgroup analyses regarding the gender and Asian dust storms. Stratified analysis was performed for male and female patients separately. The results are summarized in Supplementary File. We found that the results were similar to those for the original analysis including both male and female patients. Geographic regions with higher penalty rates had lower healthcare utilization, while the enactment of amended THPA reduced the outpatient visits in both female and male populations, while the ER visits were slightly increased in both genders (see Tables S1 and S2 of the Supplementary File). During the months with Asian dust storms, the enactment of THPA was significantly associated with a significant reduction of outpatient and ER visits, whereas during the months without Asian dust storms, the enactment of THPA was significantly associated with a significant reduction of outpatient visits, but an increased ER visits (see Table S3 of the Supplementary File).

## 4. Discussion

In this population-based study, we found that reinforcement of the smoking ban in Taiwan through the enactment of the amended THPA in 2009 was associated with a reduced risk of outpatient visits due to asthma, but with an increased risk of ER visits due to asthma in the general population. We also found that geographic regions with lower penalty rates were associated with increased outpatient and ER visits for asthma. Children under 10 years of age also had an increased risk of outpatient and ER visits for asthma.

The effect of smoking bans on health has been demonstrated in the literature [8]. The implementation of smoke-free regulations and legislation in public places such as workplaces, restaurants, and bars were shown to reduce the rate of hospitalizations in asthmatic children in a British study [7]. The amendment and enactment of the THPA in Taiwan is in line with the increasing knowledge relevant to the hazards associated with the use of tobacco. An estimated 600,000 people worldwide will die every year from second-hand smoke [17]. Moreover, the estimated annual medical costs of smoking or second-hand smoke have been reported to reach $9.6 billion in the United States [18] and £2.7 billion to £5.2 billion in the UK [19], suggesting the need for tobacco control to better manage asthma. Asthma is a serious health problem affecting all age groups; the estimated global asthma population is about 300 million, and this is expected to increase to 400 million by 2025 [20]. In the Asia-Pacific region, an increase in the prevalence of asthma and a surge in medical expenses have also been reported in recent years [21]. The adverse effects of tobacco smoke on children with asthma who currently smoke or with any exposure to environmental tobacco smoke have been reported to include an increased risk of active asthma symptoms and to account for 18% of all cases of asthma and significant medical expenditure [22]. Our study findings further provide evidence that environmental tobacco smoke significantly affects the respiratory health of children at a population scale.

As children had a significantly increased risk of both outpatient and ER visit for asthma, our findings also suggest the need for a reduction in smoking with a possible smoking ban extending to nonpublic spaces. Because the medical systems of Western counties usually require asthmatics to visit general practitioners, research based on findings from asthma clinics remains uncommon. The current study provides additional evidence of the effectiveness of reinforcing a smoking ban on outpatient visits for asthma. In addition, we showed that ER visits countrywide were not reduced, but actually slightly increased after the reinforcement of the smoking ban. Relevant reports have provided evidence of contact with smoking family members at home which may explain our findings. A study in the US investigating asthmatic children aged 8–14 years showed that the risk of an ER visit and hospitalization in these asthmatic children was correlated with the level of cotinine content in the saliva of smoking family members [23]. In addition, a study in 6 to 11 years old asthmatics in South Korea also found that smoking family members were associated with a 7.9% increased risk of asthma attacks and an increased risk of 1.049 for developing asthma [24]. In the current study, the young asthmatics accounted for about 60% of all visits to the ER due to asthma. Previous studies in Taiwan have shown an increased risk of clinic visiting after the periods of Asian dust storms [14,15,25], and the effect of Asian sand storms on asthma visits might be early within one week after the period of these storms [14,25]. Our study’s finding of increased outpatient visits during dust storm months is compatible with the literature. Our findings also showed that the effect of the smoking ban on asthma visits was more prominent during the months of Asian dust storms (Supplementary File). This might be explained by the possibility that during the dust storm periods, people tended to stay indoors, while the smoking ban might reduce the exposure to tobacco smoke for asthmatics. In addition, the probability of time lag between the implementation of smoking free law and enforcement might be reflected by the rising of penalty rates in different geographic regions across the country. As can be seen in Table 2, the trends of rising of the penalty rates were nonsynchronous among the counties. Therefore, we did not explore the effect of this potential impact of the lag. As the amended THPA in Taiwan does not ban smoking in nonpublic spaces such as homes, children are generally unable to avoid second-hand smoke from exposure to the environment, especially as they are considered to a spend considerable amount of time at home. However, direct evidence related to smoking indoors in nonpublic spaces is still lacking. In addition, we did not collect data or investigate whether smoking in public spaces was reduced by the enactment of the amended THPA. Furthermore, the penalty rate is an appropriate indicator of enforcement, as the rates shown in Table 2 showed an initial rise in most of the different geographic regions with a subsequent fall, whereas the trend of data on asthmatic visits did not show a corresponding course.

Nevertheless, we found a reduced ER visit rate in the areas with a higher penalty rate for violating the smoking ban after the enactment of the THPA. Our findings are comparable with research across other public health issues. A study in Texas on alcohol control policies reported that an increase in alcohol taxation led to a decline in alcohol sales, which in turn reduced the rate of drunk driving [26]. Another study in Taiwan on drunk driving showed that penalty rates were inversely correlated with accident rates due to drunk driving [27]. Therefore, this study may provide reasonable and valuable insights into further promotion of the smoking ban in the future regarding asthma care. The generalizability of our study findings may be applicable to similar health systems with adequate health insurance coverage and reimbursement policies for asthma with regards to outpatient and ER visits.

There are several limitations to this study. First, this was an observational study with no control population or group. However, a prospective controlled study or quasi-experimental study would be challenging for this issue, as the tobacco ban includes the general population. Second, the NHIRD provides useful information about patient characteristics and clinical information, however limited information is available with regards to smoking status or exposure to environmental tobacco smoke. The NHIRD also lacks data on disease severity and lung function status related to asthma. Third, our data showed that there was a variation in the penalty rate for violating the smoking ban across different geographic regions of Taiwan, which may also suggest the limited efficacy of the smoking ban and concerns over the sustainability of the THPA. Fourth, we did not investigate inpatient healthcare utilization. A report in the USA reported a ratio of cases of hospitalization to ER visits of about 1:4, which may infer relative healthcare utilization [10]. Therefore, even though our study did not cover inpatient healthcare utilization, our findings may explain the majority of healthcare resource utilization in Taiwan. Fifth, we used the number of visits and rates of outpatient and ER visits for asthma instead of the costs and expenses related to asthma care, and we did not explore the costs related to the enactment of the THPA and the public announcement and practice of imposing penalties for smoking violations. This may limit the estimation of the financial impact of enacting the smoking ban at the population level. Sixth, while hospitalization rates are still important, the current sample of this study probably missed the cases of more severe asthma with hospitalization as the main healthcare utilization. Therefore, the effect of cases with hospitalizations was not calculated, which might reflect an outcome misclassification bias rather than a true reduction of asthma visits.

## 5. Conclusions

In conclusion, the enactment of the amended THPA in Taiwan was associated with a reduced risk of outpatient visits for asthma, but an increased risk of ER visits for asthma. As we found that children were affected most, with a significantly increased risk of both outpatient and ER visits, we recommend strengthening the penalties for smoking in public spaces further and reducing indoor smoking in nonpublic spaces to reduce healthcare utilization.

## Figures and Tables

**Table 1:** Outpatient and emergency department visits for asthma, 2005–2013.

Variable	Outpatient Visits	Emergency Department Visit
All	4,427,048 (100%)	160,218 (100%)
*Gender*		
Male	2,543,475 (57.5%)	99,501 (62.1%)
Female	1,883,573 (42.5%)	60,717 (37.9%)
*Age group (years)*		
0–9	2,083,841 (47.1%)	75,048 (46.8%)
10–19	427,116 (9.7%)	22,517 (14.1%)
20–59	60,530 (1.4%)	2,617 (1.6%)
60–69	619,527 (14.0%)	16,741 (10.5%)
≧70	1,236,034 (27.9%)	43,295 (27.0%)
Mean (SD)	34.11 (33.56)	32.17 (33.35)
*Enactment of the amended THPA*		
Before (2005–2008)	1,919,687 (43.4%)	71,211 (44.4%)
After (2009–2013)	2,507,361 (56.6%)	89,007 (55.6%)
*Penalty Rate (2009–2013)*		
High	1,011,696 (40.4%)	31,474 (35.4%)
Medium	797,259 (31.8%)	29,527 (33.2%)
Low	698,406 (27.9%)	28,006 (31.5%)

THPA, Tobacco Hazards and Prevention Act; SD, standard deviation.

**Table 2:** Penalty rates according to geographic distribution of cities and counties.

City/County	2009 Rate (‰)	2010 Rate (‰)	2011 Rate (‰)	2012 Rate (‰)	2013 Rate (‰)	Average Rate (‰)
*High*						
Hsinchu City	31.5	63.4	32.9	9.9	5.7	28.7
Kaohsiung City	5.3	9.4	36.3	10.9	30.3	18.4
New Taipei City	35.4	30.4	21.2	1.1	2.6	18.1
Taoyuan County	24.4	14.4	10.9	4.0	2.0	11.2
*Medium*						
Taichung City	10.0	10.9	15.6	6.3	6.8	9.9
Yunlin County	9.8	18.2	10.3	6.7	3.2	9.6
Pingtong County	5.9	13.9	16.9	4.7	4.4	9.2
Jinmen County	0.6	10.3	0.8	7.9	25.5	9.0
Tainan City	7.4	8.6	18.8	5.3	4.3	8.9
Keelung City	1.0	11.0	13.7	7.7	9.8	8.6
Hualien County	4.5	9.3	11.6	3.0	13.8	8.5
Hsunchu County	13.6	16.7	1.9	0.4	1.0	6.7
Lianjiang County	7.8	2.5	0.0	4.2	18.2	6.6
Miaoli County	3.2	8.6	4.3	6.5	10.1	6.5
Chiayi County	5.4	12.9	5.9	2.4	4.1	6.2
*Low*						
Taipei City	2.5	5.6	11.2	3.9	2.8	5.2
Chiayi City	0.4	2.4	9.6	2.5	5.9	4.2
Yilan County	2.0	3.1	3.2	3.3	2.6	2.9
Taitung County	2.8	5.2	1.3	1.0	2.8	2.6
Changhua County	2.0	4.0	4.2	0.9	1.3	2.5
Nantou County	1.1	5.0	1.5	0.8	1.5	2.0
Penghu County	0.5	1.0	0.5	0.0	0.3	0.5

Data source: https://www.mohw.gov.tw/dl-55656-26dd30b1-7418-4b7b-9a9a-b2ca460913d5.html.

**Table 3:** Crude rates of outpatient and emergency department visits before and after the enactment of the THPA.

Variable	Outpatient Visits Rate (‰)			Emergency Department Visits Rate (‰)		
	Before (2005–2008)	After (2009–2013)	Difference	Before (2005–2008)	After (2009–2013)	Difference
*All*	1.75	1.80	0.05	0.06	0.06	-
*Gender*						
Male	2.01	2.04	0.03	0.08	0.08	-
Female	1.48	1.56	0.08	0.05	0.05	-
*Age group (years)*						
0–9	7.01	9.64	2.63	0.27	0.33	0.06
10–19	1.11	1.40	0.29	0.06	0.07	0.01
20–59	0.05	0.03	−0.02	0.00	0.00	-
60–69	4.41	2.67	−1.74	0.12	0.07	−0.05
≧70	7.30	6.51	−0.79	0.25	0.23	−0.02
*Penalty Rate*						
High		1.85			0.06	
Medium		1.55			0.06	
Low		2.12			0.08	

THPA, Tobacco Hazards and Prevention Act.

**Table 4:** Poisson regression analysis of the risk of an outpatient visit and emergency department visits for asthma: the effect of smoking ban penalty.

Variable	Outpatient Visits		Emergency Room Visits	
	RR	95% CI	RR	95% CI
*Penalty rate*				
*Low*	1.00	reference	1.00	reference
*Medium*	0.79 ***	0.79–0.80	0.68 ***	0.68–0.69
*High*	0.91 ***	0.91–0.92	0.75 ***	0.74–0.76
*Enactment of amended THPA*				
*Before*	1.00	Reference	1.00	reference
*After*	0.98 ***	0.98–0.99	1.07 ***	1.05–1.09
*Year trend after*	0.99 ***	0.99–1.00	1.00	1.00–1.00
*Dust Storm_*	1.03 ***	1.02–1.03	1.02	1.00–0.03

THPA, Tobacco Hazards and Prevention Act; RR, relative risk; CI, confidence interval, *** *p* < 0.0001.

## Supplementary Materials

The following are available online at https://www.mdpi.com/1660-4601/16/20/3950/s1. ‘Supplementary File.docx’: additional information regarding subgroup analysis for gender and Asian dust storms with stratified analysis for healthcare utilization for the outpatient and emergency settings.
