# Supplementary material for: Reinforcement of Tobacco Control and Reduction in Medical Utilization for Asthma in Taiwan: A Population-Based Study
Source: Int J Environ Res Public Health. 2019 Oct 17;16(20):3950. doi: 10.3390/ijerph16203950 (PMC6844081; doi:10.3390/ijerph16203950)
Supplement: Supplementary file 1 [file ijerph-16-03950-s001.pdf]

Supplementary materials

Reinforcement of tobacco control and reduction in medical utilization for asthma in Taiwan: A population-based study

Huang-Ju Liang [1,4], Ming-Jiuan Wu [2], Jih-Shuin Jerng [3,4,*] and Chiang-Hsing Yang [1]

[1] Department of Health Care Management, National Taipei University of Nursing and Health Sciences, Taipei 108, Taiwan; 020096@ntuh.gov.tw (H.-J.L.); yangch@ntunhs.edu.tw (C.-H.Y.)

[2] Center for Quality Management, National Taiwan University Hospital, Taipei 100, Taiwan

[3] Department of Business and Management, Ming Chi University of Technology, New Taipei City 243, Taiwan; mjgrace@mail.mcut.edu.tw

[4] Department of Internal Medicine, National Taiwan University Hospital, Taipei 100, Taiwan

* Correspondence: jsjerng@ntu.edu.tw; Tel.: +886-2-23123456

S1. Subgroup analysis

S1.1. The effect of gender

We performed stratified analysis regarding the gender for subgroup analysis for male and female patients separately. We found that the results were similar to those for the original analysis, including both male and female patients. Table S1 shows the results of stratified analysis for male patients included in this study. Geographic regions with medium penalty rates (RR: 0.80, 95% CI: 0.80-0.80) and high penalty rates (RR: 0.91, 95% CI: 0.90-0.91) had lower outpatient healthcare utilization. Similar trends of reduced healthcare utilization were also found at the emergency setting (RR: 0.69, 95% CI: 0.67-0.70 for medium penalty-rate, and HR: 0.75, 95% CI: 0.73-0.76 for high penalty-rate regions). The enactment of amended THPA reduced the outpatient visits (RR: 0.98, 95%CI: 0.98-0.99), but the ER visits were slightly increased in male gender (RR: 1.09, 95% CI: 1.06-1.11).

Table S1. Stratified analysis of the risk of an outpatient visit and emergency department visits for asthma in male patients: effect of smoking ban penalty.

Variable	Outpatient visits		Emergency room visits	
	**RR**	**95% CI**	**RR**	**95% CI**
Penalty rate				
Low	1.00	reference	1.00	reference
Medium	0.80***	0.80-0.80	0.69***	0.67-0.70
High	0.91***	0.90-0.91	0.75***	0.73-0.76
Enactment of amended THPA				
Before	1.00	Reference	1.00	reference
After	0.98***	0.98-0.99	1.09***	1.06-1.11
Year trend after	1.01***	1.01-1.01	1.00	1.00-1.00

THPA, Tobacco Hazards and Prevention Act; RR, relative risk; CI, confidence interval
p*<0.05, p**<0.005, p***<0.0001

Table S2 shows the results of the stratified analysis for female patients included in this study. Geographic regions with medium penalty rates (RR: 0.79, 95% CI: 0.78-0.79) and high penalty rates (RR: 0.93, 95% CI: 0.92-0.93) had lower outpatient healthcare utilization. Similar trends of reduced healthcare utilization were also found at the emergency setting (RR: 0.68, 95% CI: 0.67-0.70 for medium penalty-rate, and RR: 0.75, 95% CI: 0.73-0.76 for high penalty-rate regions). The enactment of amended THPA reduced the outpatient visits (RR: 0.98, 95% CI: 0.97-0.98), but the ER visits were slightly increased in female gender (RR:1.04, 95% CI: 1.00-1.07).

Table S2. Stratified analysis of the risk of an outpatient visit and emergency department visits for asthma in female patients: effect of smoking ban penalty.

**Variable**	**Outpatient visits**		**Emergency room visits**	
	**RR**	**95% CI**	**RR**	**95% CI**
Penalty rate				
Low	1.00	reference	1.00	reference
Medium	0.79***	0.78-0.79	0.68***	0.67-0.70
High	0.93***	0.92-0.93	0.75***	0.73-0.76
Enactment of amended THPA				
Before	1.00	Reference	1.00	reference
After	0.98***	0.97-0.98	1.04	1.00-1.07
Year trend after	1.00***	1.00-1.00	1.00	1.00-1.00

THPA, Tobacco Hazards and Prevention Act; RR, relative risk; CI, confidence interval
*p<0.05, **p<0.005, ***p<0.0001

S1.2. The effect of Asian dust storms

We performed stratified analyses of asthmatic visit rates to see the effect of the smoking ban, based on the periods with or without Asian dust storms. Table S3 shows that during the dust storm months, the rates of outpatient visits (RR: 0.89, 95% CI: 0.87-0.91) and emergency visits (RR: 0.71, 95% CI: 0.65-0.78) were both reduced after the enactment of smoking ban (Table S3). During the months without Asian dust storms, the rate of outpatient visits was reduced after the enactment of smoking ban (RR: 0.98, 95% CI: 0.98-0.98), but the rate of emergency visits was increased (RR: 1.08, 95% CI: 1.05-1.10) (Table S3).

Table S3. Stratified analysis of the risk of an outpatient visit and emergency department visits for asthma in Dust Storm and Dust Storm-free patients: effect of smoking ban.

**Subgroup**	**Variable**	**Outpatient visits**		**Emergency room visits**	
		**RR**	**95% CI**	**RR**	**95% CI**
	Enactment of amended THPA				
Dust Storm (+)	*Before*	1.00	Reference	1.00	reference
	After	0.89***	0.87-0.91	0.71***	0.65-0.78
	Year trend after	1.01***	1.01-1.01	1.01***	1.01-1.02
	Enactment of amended THPA				
Dust Storm (-)	*Before*	1.00	Reference	1.00	reference
	After	0.98***	0.98-0.98	1.08***	1.05-1.10
	Year trend after	1.01***	1.01-1.01	1.01***	1.01-1.01

THPA, Tobacco Hazards and Prevention Act; RR, relative risk; CI, confidence interval
*p<0.05, **p<0.005, ***p<0.0001
